# Characterization of potential donors of multiple organs and tissues reported in Maranhão from 2009 to 2012

**DOI:** 10.1186/cc12648

**Published:** 2013-06-19

**Authors:** TM Correa, G Castro, JS da Silva, JN Bacelar

**Affiliations:** 1 Hospital Centro Médico, Monte Castelo, São Luís, MA, Brazil

## Introduction

Thousands of lives worldwide are improved annually with transplant and donation of organs and tissues. Human organ transplant was one of the greatest medical advances in the 20th century, with a success rate between 80 and 90%. The Brazilian National Transplantation System coordinates and regulates perhaps the largest public transplantation program worldwide. However, family refusal represents the biggest obstacle to the donation process. It is one of the main factors responsible for the shortage of organs and tissues for transplantation. The objectives of this study were to characterize potential donors reported to the Central of Notification, Captation and Distribution of Organs and Tissues in Maranhão (CNCDO-MA) between January 2009 and September 2012, to evaluate the frequency of potential donors converted into effective donors, and to identify the captured organs in effective donors.

## Methods

A descriptive, retrospective study and quantitative analysis from CNCDO-MA reports, using a standardized data collection form. The inclusion criteria was suspected brain death (BD), with at least the first clinical examination for BD protocol performed - potential donor, notified to CNCDO-MA between January 2009 and September 2012. The exclusion criteria were the notifications in which the cause of death was unknown or supplementary test results were inconsistent with BD.

## Results

A total of 345 notifications were analyzed. The majority were male (63.2%), with an average age of 37.85 (± 18.47) years. The most incident causes of BD were traumatic brain injury (39.7%) and hemorrhagic stroke (36.2%). Arteriography was the predominant supplementary examination (14.5%) to complete the BD protocol. The potential donors converted into effective donors were 14.8%. The amount of collected organs in effective donors was 50 corneas, 29 kidneys, six hearts and one liver.

## Conclusion

The notification and donation of organs and tissues rates are lower in Maranhão than in Brazil. The integration of multidisciplinary teams is necessary for the responsibility of maintaining potential donors and mandatory BD notification, regardless of the chance of organ donations, which can improve the rates in our state.

